# In the Eye of the Beholder: Reduced Threat-Bias and Increased Gaze-Imitation towards Reward in Relation to Trait Anger

**DOI:** 10.1371/journal.pone.0031373

**Published:** 2012-02-17

**Authors:** David Terburg, Henk Aarts, Peter Putman, Jack van Honk

**Affiliations:** 1 Department of Psychology, Utrecht University, Utrecht, The Netherlands; 2 Department of Psychology, Leiden University, Leiden, The Netherlands; 3 Department of Psychiatry and Mental Health, University of Cape Town, Cape Town, South Africa; Royal Holloway, University of London, United Kingdom

## Abstract

The gaze of a fearful face silently signals a potential threat's location, while the happy-gaze communicates the location of impending reward. Imitating such gaze-shifts is an automatic form of social interaction that promotes survival of individual and group. Evidence from gaze-cueing studies suggests that covert allocation of attention to another individual's gaze-direction is facilitated when threat is communicated and further enhanced by trait anxiety. We used novel eye-tracking techniques to assess whether dynamic fearful and happy facial expressions actually facilitate automatic gaze-imitation. We show that this actual gaze-imitation effect is stronger when threat is signaled, but not further enhanced by trait anxiety. Instead, trait anger predicts facilitated gaze-imitation to reward, and to reward compared to threat. These results agree with an increasing body of evidence on trait anger sensitivity to reward.

## Introduction

Primate and especially human social interaction depend heavily on non-verbal communication with the eyes [1]. The elongated width and extreme whiteness of the sclera are indeed unique features of the human eyes, argued to have evolved to facilitate such social communication [2]. Interestingly, following the gaze of others is reflexive and can therefore be regarded as adaptive behavior crucial to survival [3], [4]. Detection of, and attending to threat are evidently adaptive behaviors. Accordingly, facial expressions [5] as well as gaze-direction [6] are processed automatically and preconsciously.

Humans and other primates actively follow observed gaze-shifts [1], [7], but although it would provide a unique insight in reflexive and adaptive social behavior, it has not yet been experimentally studied how facial expressions influence these gaze imitations. It is however known that faces with averted gaze are labeled faster and more often as fearful, while the opposite holds for happy faces [8], [9]. Moreover, facial expressions can give relevance and meaning to the gaze-shift with regard to mental state and social environment [10]. For example, a happy gaze-shift may signal a potential reward, while a frightened gaze-shift can alert for potential threat. Although the latter is often considered to be more crucial to survival [3], studies on attentional cueing by observed gaze-shifts, or gaze-cueing, have struggled to find general effects of facial expression [11]. More recent studies revealed however a threat bias in gaze-cueing by fearful faces [12], [13], but there is also evidence that this is exclusive to high anxious individuals [14], [15]. Studies using more ecologically valid dynamic facial stimuli confirmed that the threat bias in gaze-cueing is strongest in high anxious individuals, but also showed reliable general effects of facilitated gaze-cueing by fearful compared to happy facial expressions [16]–[18].

Although these studies provide valuable information on gaze-cueing of covert attention, their generalizability to real-life social behavior is limited because participants are instructed to refrain from making gaze-movements, and the measures of interest (e.g. button-presses and symbol-identification) are non-adaptive behavioral responses. The natural response to a gaze-shift is however to actively follow it, which is an adaptive feature of primate [1] and human [7] behavior, already observed in new-borns between 1 and 3 days old [19].

Studies on overt gaze-cueing, or ‘gaze-imitation’, in adults are scarce, but confirm that the preparation of gaze-imitation saccades is reflexive [4]. Unlike reflexive covert shifts of attention, however, the actual execution of these eye-movements can be inhibited and are therefore prone to top-down modulation [3], [20]. Importantly, although threat detection in gaze-cueing paradigms is enhanced in relation to anxiety [14], [15], anxiety is also strongly related to threat avoidance [21], particularly in relation to eye movement responses [22]. The anxious priority for threat in reflexive gaze-cuing might therefore not simply be applicable to the overt case.

In relation to trait anger, on the other hand, no such threat avoidance should be expected. Moreover, trait anger apparently is highly predictive for social aggression, which is marked by reduced sensitivity to the victim's fearful expression (see [23] for a review). In strong agreement, trait anger is related to reduced amygdala reactivity when perceiving fearful faces [24]. Additionally, trait anger has repeatedly been linked to reward-sensitivity and approach motivation [25], [26]. Accordingly, the motivational drive to follow a gaze-shift might be decreased for fearful, but increased for happy cues, because the latter signals a peripheral reward.

Affective modulation of overt gaze-imitation by cues of threat and reward has not yet been experimentally studied. Therefore, we developed a new gaze-imitation task that closely resembles a situation wherein someone actively shifts gaze to a rewarding or threatening location. Participants watched video-clips of faces shifting gaze in a happy or fearful manner, and responded by gazing as fast as possible to a target appearing in the gaze-signaled, or opposite, location. This paradigm allowed us to assess whether imitative gaze-shifts are facilitated towards threat or reward and how this interacts with personality traits of anger and anxiety.

We expected faster gaze-allocation when an observed gaze-shift was imitated and further facilitation when threat was signaled with a fearful expression. Furthermore, in light of the enhanced threat detection [18] and threat-avoidance [21], [22] in relation to anxiety, the positive relations between trait anxiety and covert fear-gaze cueing [13]–[15], [18] might not be observed here. A happy gaze-shift, on the other hand, signals a potential peripheral reward. Since trait anger is associated with increased reward sensitivity [25], [26], we predict that individuals high in trait anger are relatively more motivated to follow a happy gaze-shift, which should reduce the expected priority for gaze-imitation towards threat over reward.

## Methods

### Ethics Statement

The research reported in this article involves healthy human participants, and does not utilise any invasive techniques, substance administration or psychological manipulations. Therefore, compliant with Dutch law, this study only required, and received approval from our internal faculty board (Human Biopsychology and Psychopharmacology) at Utrecht University. Furthermore, this research was conducted, and written informed consent of each participant obtained, according to the principles expressed in the Declaration of Helsinki.

### Participants and Procedure

Twenty healthy volunteers (all students, age-range 18–25 years, 9 female) received course credit or a monetary reward to participate in the experiment. Stimuli and design were adapted from Putman and colleagues [18] and consisted of video-clips of centrally presented faces changing rapidly (120 ms) from neutral to either happy or fearful, while the eyes simultaneously moved from central to peripheral gaze (left and right). The final frame was maintained for an additional 80 ms, after which the face disappeared and in 2/3 of the trials, a target appeared either to the left or right (10° visual angle) of the face.

For the video-clips 8 different actors (4 female), with 2 emotions (happy and fearful) and 2 gaze-directions (left and right), were used [27], [28], making 32 unique stimuli (see [18] for further details). These were presented 6 times each; twice with a target at the same location as the gaze-shift (valid trial), twice with a target at the opposite location to the gaze-shift (invalid trial) and twice with no target to avoid habitual saccade preparation (catch trial). These made a total of 192 trials, counterbalanced for emotion and condition, and presented in random order. Preceding the task, nine trials were presented for practice, using the same stimuli with gaze-, but without shift of emotion.

**Figure 1:**
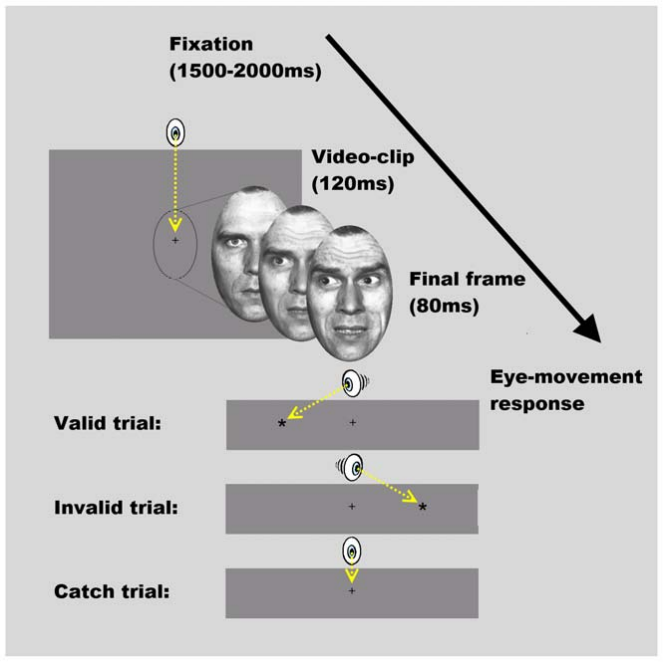
Visual representation of the gaze-imitation task. After gaze-fixation participants watched video-clips wherein faces fluently shifted from neutral to fearful or happy expressions, while the eyes shifted from center to left or right. Participants were instructed to allocate their gaze as fast as possible to the target that appeared on the left or right side of the screen when the clip ended. One-third of the trials was valid (target in same location as stimulus gaze-shift), one-third invalid (target in opposite location of stimulus gaze-shift) and one-third catch (without target, thus without eye-movement response). The example stimulus shown here was adapted from the Pictures of Facial Affect database [27].

Participants were instructed to shift their gaze towards the target, and were explicitly, and correctly, informed that gaze-direction of the presented face did not predict target appearance or location. Responses were made with a shift of gaze to the target, which disappeared when the eye-track computer detected that the target was reached. Stimulus presentation commenced when the participant gazed at a fixation-cross, positioned where the eyes of the subsequently presented faces would appear, for a random time between 1000 ms and 1500 ms to avoid timing habituation. During the catch trials, wherein no target appeared, gaze had to be maintained at the fixation position until start of the next trial (see **Figure 1** for a visual representation of the task). Beforehand, participants completed trait anxiety and anger (STAI/STAS) questionnaires [29], [30].

### Apparatus and analyses

For the present study we are primarily interested in gaze-shifts, as this is the most natural way of overt orienting. A gaze-shift consists of an eye-movement, and a simultaneous, but small, head-movement [31], which is restricted by most eye-track systems using head-fixation. The gaze-imitation task was therefore presented, and gaze-data recorded, using a Tobii-1750 binocular eye-tracker with integrated TFT-display, 8 ms response time, 50 Hz sampling-rate and 0.5° accuracy [32]. With this eye-track system head-fixation is not necessary, which allows for relatively unrestricted gaze responses.

Latency of the gaze-shifts was defined as the time between onset of, and first gaze-point within 1° of the target. Trials with latencies shorter than 100 ms or longer than 1200 ms (0.4%) were removed from analysis. Mean latencies were computed for all 4 conditions (threat/reward×valid/invalid), and were used in three analysis steps. First, we assessed overall and emotion-specific gaze-imitation effects. Thereto, mean latencies were entered as within-subject variables in a 2×2 repeated-measures ANOVA, followed by paired-samples t-tests.

Second, we assessed the emotion-specific influence of the personality characteristics STAI and STAS on gaze-imitation. STAI and STAS scores were correlated with gaze-imitation biases computed for both emotions separately by subtracting the average latencies on the valid from the invalid trials. These contrasts provide a reliable measure of gaze-imitation, because if gaze-imitation is a reflexive mechanism, this would affect both conditions in opposite direction; i.e. gaze-shifts will be facilitated in the valid trials, and delayed in the invalid trials. Furthermore, these bias-scores represent emotion-specific indices of gaze-imitation without confounding effects of between-subjects variability in overall reaction speed, whereby higher values represent stronger effects of gaze-imitation.

Third, we assessed how STAI and STAS influenced gaze-imitation towards reward compared to threat. In classic attentional-cueing [33], and covert gaze-cueing experiments [18], such top-down modulation is often described in terms of engagement and disengagement. The first applies to the valid trials only, and is a measure of how fast attention is directed towards a peripheral target, whereas the second applies to the invalid trials as a measure of how fast one can disengage attention from a peripheral location. For direct assessment of the effect of personality characteristics on the difference between gaze-imitation towards threat and reward, however, we are primarily interested in how the imitative gaze-shift (i.e. the engagement component) is modulated, because this constitutes the top-down influence on actual gaze-imitation. Moreover, the disengagement component, or the shift of gaze in the opposite direction to an observed gaze-shift, involves suppression and inversion of the initial gaze-imitation reflex. In other words, while disengagement in gaze-cueing studies is a purely attentional mechanism, in a gaze-imitation task it would involve inhibition of reflexive motor-responses [34]. A reliable assessment of between-emotion differences in disengagement would therefore involve in-depth saccade analysis to identify these, likely small, erroneous saccades. The gain of minimal movement restriction, provided by the use of the Tobii-1750 eye-tracker, came however with the cost of a relatively low sampling-rate of gaze-data, which does not allow for such analyses. Therefore we assessed the top-down influence on gaze-imitation towards threat compared to reward only in the valid condition. STAI and STAS were thereto correlated with threat/reward bias scores computed by subtracting the average latencies on the valid-fear trials from the valid-happy trials. Thus, higher values represent a gaze-imitation bias for threat relative to reward.

In sum, we first assessed overall gaze-imitation and the difference between gaze-imitation towards threat and reward. Next, we assessed modulation of gaze-imitation by STAI and STAS through contrasting valid and invalid trials for each emotion. Finally, we assessed the effect of these two personality traits on the actual affective modulation of gaze-imitative gaze-shifts by computing their correlation with the contrast of threat and reward trials in the valid condition. All reported statistics are conducted with two-sided α = 0.05.

## Results

**Figure 2:**
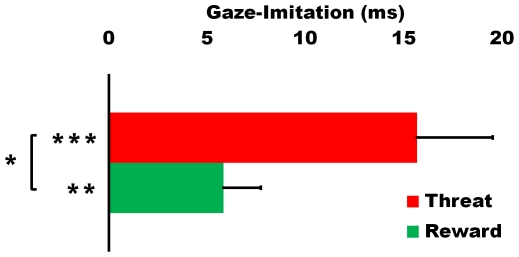
Gaze-imitation effects for the threat (fearful faces) and reward (happy faces) conditions. Values represent mean latencies of gaze-allocation in invalid minus valid trials. The gaze-imitation effect is significant in both conditions and significantly stronger in the threat condition. Error-bars represent SEM. * = p<.05. ** = p<.01. *** = p<.001.

Mean latencies of gaze-allocations for all four conditions are shown in **Table 1**. We found a significant effect of validity (F(1,19) = 19.253, p<.001, η_p_^2^ = .503), and a significant interaction of validity and emotion (fear/happy) showed that the validity effect, or gaze-imitation, was reliably stronger in the fear compared to happy condition (F(1,19) = 7.680, p<.05, η_p_^2^ = .288, see **Table 1** and **Figure 2**). Separate paired-sample t-tests confirmed reliable gaze-imitation effects for both the fearful (16 ms faster in valid trials, t(19) = 4.084, p<.001) and happy (6 ms faster in valid trials, t(19) = 3.083, p<.01) conditions. Furthermore, the main effect of emotion was significant for the valid condition (8 ms faster in fearful trials, t(19) = 2.109, p<.05), but not for the invalid condition (2 ms slower in fearful trials, t(19) = −.604, p = .553). This confirms that in the valid trials, where the observed gaze-shifts are imitated, gaze-shifts were faster when the observed gaze-shift was accompanied with a fearful expression.

**Table 1:** Mean latencies (with standard deviation) of gaze-allocation for each condition in the gaze-imitation task.

	Threat (fearful face)	Reward (happy face)
**Valid**	281 (27) ms	289 (32) ms
**Invalid**	297 (34) ms	295 (31) ms

**Figure 3:**
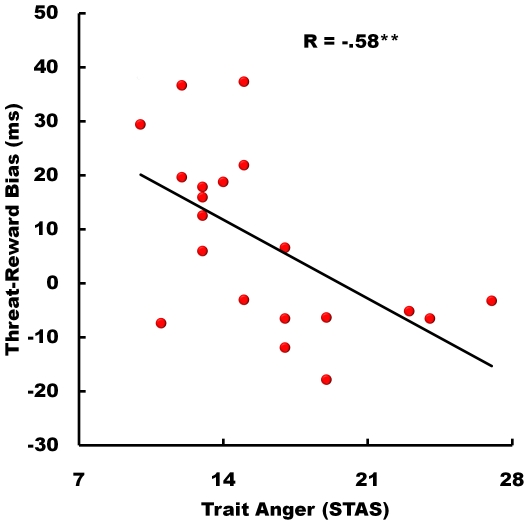
Linear relation of trait anger (STAS) with the threat-reward bias. High values represent a stronger gaze-imitation effect towards threat.

The correlational analysis showed that trait anxiety (STAI) and trait anger (STAS) were not significantly related in our subject sample (R = .19, p = .416). Furthermore, STAI was not related to gaze-imitation, as indexed by the contrast of invalid minus valid trials, in the fear (R = −.08, p = .737) and happy (R = .15, p = .519) conditions. STAS was as predicted significantly related to increased gaze-imitation in the happy (R = .54, p<.05), but not in the fear (R = .23, p = .324) condition. Finally, as predicted, STAS was strongly related to a reduced fear/happy bias in the valid condition (R = −.58, p<.01, see **Figure 3**), while for STAI there was no significant relation (R = .16, p = .492). In sum, gaze-imitation is not directly modulated by STAI, but STAS is associated with greater gaze-imitation towards reward as signaled by happy facial expressions, and with a reduced gaze-imitation bias towards threat as signaled by fearful compared to happy facial expressions.

## Discussion

In this study we show that allocation of gaze is faster when the gaze-shift of someone else is imitated. Moreover, when the observed gaze-shift is accompanied with a dynamic fearful expression, which communicates a peripheral threat, the gaze-imitation effect is stronger than when peripheral reward is signaled with dynamic happy gaze-shifts. As predicted, this threat-bias was strongly reduced in relation to heightened trait anger, but unrelated to trait anxiety.

Firstly, these results replicate the findings of Ricciardelli and colleagues [4], who found facilitated allocation of gaze in the direction of observed (neutral) gaze-shifts. Secondly, the threat-bias in gaze-imitation concurs with the literature on gaze-cueing, and is arguably an adaptive reflex. The biological underpinnings of this reflex might be found in the amygdala's involvement in the processing of both gaze and emotional expression. Direction of gaze is processed in the superior temporal sulcus (STS), which projects both to intraparietal areas for subsequent allocation of attention, as well as to the amygdala [1], [3]. Moreover, the amygdala is automatically activated by threat, and fearful faces in particular [35], and has both direct and indirect influence on the allocation of attention towards threat [36]. STS-amygdala interactions might therefore underlie the integration of affective value and gaze-direction [10], and thus the present reflexive modulation of gaze in response to threat.

On top of this emotional modulation, we show here that the gaze-imitation bias for threat compared to reward is reduced in relation to trait anger. As mentioned in the introduction, trait anger has repeatedly been related to increased reward sensitivity [25], [26]. A happy gaze-shift as signal of potential peripheral reward may therefore carry high motivational value for those high in anger, which might have resulted in the here found increase of gaze-imitation towards reward in relation to trait anger. It must however be noted that, based on the present data, we cannot entirely exclude that trait anger also reduces gaze-imitation towards threat. Indeed, trait anger is associated with reduced amygdala activity when perceiving fearful faces [24], and reduced sensitivity for fearful facial expressions, which is argued to underlie social aggression [23]. The present data are however in favor of increased reward-sensitivity in relation to trait anger, and we therefore assume that the trait anger shift from threat to reward in gaze-imitation is driven by angry individuals imitating happy gaze more strongly, thereby reducing the general imitation bias for fearful gaze-shifts.

Our results furthermore show that trait anxiety has no direct relation to the emotional modulation of gaze-imitation. In the light of recent evidence that covert gaze-cueing towards threat is enhanced in relation to anxiety [12], [13], [18], and sometimes even exclusive to anxiety [14], [15], this is an intriguing finding. Crucially, Putman and colleagues [18] confirmed, with the exact same stimuli and design in a study on covert gaze-cueing (i.e., with button-press on target-detection), enhancement of the threat-bias in relation to trait anxiety. Apparently anxiety facilitates target-detection when an observed gaze-shift indicates that it might be a threat, but does not facilitate overt responding towards the threat. Importantly, in the present paradigm covert target-detection always precedes the actual gaze-shift, and it therefore seems that the increase in threat-detection speed in relation to anxiety is somehow counteracted during the subsequent overt response.

These contrasting effects might be explained by the fact that overtly gazing at a threat can be distressing, which is an essential feature of the vigilance-avoidance hypothesis of anxiety. The vigilance-avoidance hypothesis [21], predicts that increased vigilance facilitates detection of threat in anxious individuals, but that this threat is subsequently defensively avoided to reduce internal distress. Indeed, as already noted, the overt gaze-imitation reflex can be inhibited [20], thus the increased speed of covert target-detection when a gaze-shift indicates that it is a possible threat , might be reflexively counteracted by anxious avoidance mechanisms. Speculatively, in the case of gaze-imitation, anxious individuals put the attentional system in reverse after a threat has been detected, in order to avoid confrontation, and reduce internal distress. A limitation of the present study is however that we did not assess target-detection and overt responding separately. Whether gaze-shifts towards threat are indeed counteracted, or maybe simply not affected by anxiety, is therefore something that should be tested in future research.

Another limitation of the present study is that the design did not allow for a neutral baseline measure. Although the correlational analysis shows us that the reduced threat-bias in relation to trait anger is most likely the result of increased gaze-imitation towards reward, we cannot exclude that gaze-imitation towards threat might also be reduced. Both interpretations are supported in the literature [23]–[26], and future research on gaze-imitation should therefore address this issue.

In summary, allocation of gaze is reflexively facilitated when an observed gaze-shift is imitated. When someone gazes away fearfully, signaling a potential threat, this gaze-imitation effect is stronger. Moreover, we provide evidence that trait anger shifts this threat-bias towards relatively stronger imitation of happy facial cues; i.e. a shift in the sensitivity for threat towards reward. Additionally, in line with the vigilance-avoidance hypothesis we speculate that trait anxiety induces conflict between facilitated covert threat-detection and overt threat-avoidance. Finally, the study of actual gaze-behavior appears to be an ecologically valid method to promote the understanding of the mechanisms behind real-life gaze following behavior in relation to anxiety and anger. Taken together with the large body of work accumulated in recent years on covert attentional mechanisms, the study of interactive overt social gaze-behavior can importantly contribute to psychology and neuroscience.
